# Cerebral Arteriovenous Malformation Deep Draining Veins Not Observed on Preoperative Angiography Identified on Postoperative Angiography

**DOI:** 10.7759/cureus.16410

**Published:** 2021-07-15

**Authors:** Austin Gamblin, Sarah Nguyen, Vance Fredrickson, Ramesh Grandhi, William T Couldwell

**Affiliations:** 1 Neurosurgery, University of Utah, Salt Lake City, USA

**Keywords:** arteriovenous malformation, microsurgery, postoperative angiography, case report, spetzler-martin grade

## Abstract

Postoperative digital subtraction angiography (DSA) is the gold standard for establishing a cure of an arteriovenous malformation (AVM) after treatment. The incidence of residual AVM identified on postoperative DSA ranges from 1.8 to 11%. Although this is important for finalizing the treatment of AVMs, postoperative DSA rarely shows new findings that were not previously identified on preoperative imaging. We present a unique case where we identified residual AVM nidus on immediate postoperative DSA that drained into two deep veins that were not evident on preoperative DSA and increased the AVM grade from Spetzler-Martin grade II to III. To our knowledge, this finding has not been previously reported in the literature. We resected the residual AVM nidus identified on postoperative DSA, leading to an angiographic cure. The patient demonstrated a postoperative right-sided supplementary motor area syndrome that resolved over time with therapy. She made a complete functional recovery by her one-month follow-up appointment.

## Introduction

Postoperative digital subtraction angiography (DSA) is considered the gold standard for establishing a cure of a cerebral arteriovenous malformation (AVM) [1,2]. Postoperative DSA can identify a surgical cure or may demonstrate residual AVM and guide further treatment [3]. It is difficult to identify the true incidence of residual AVMs on postoperative DSA because the reported incidence varies from 1.8 to 11% [3,4]. These studies did not report residual findings on postoperative DSA that resulted in a change in the Spetzler-Martin grade (S-M grade) [4].

We present a unique case where we identified residual AVM on DSA after resection. The residual nidus drained into two deep veins that were not evident on preoperative DSA, increasing the AVM grade from S-M grade II to III. To our knowledge, this finding has not been previously reported in the literature.

## Case presentation

**Figure 1:**
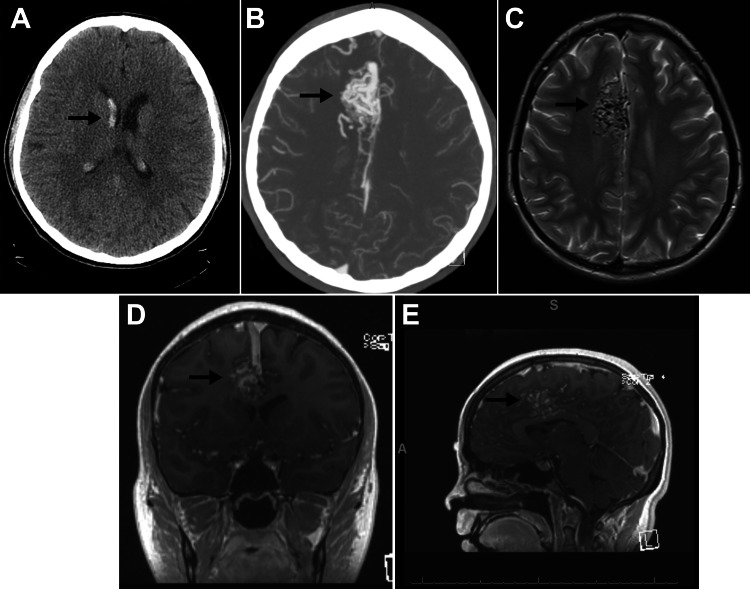
Imaging of intraventricular hemorrhage and AVM. (A) Axial noncontrast CTH demonstrates intraventricular hemorrhage primarily in the right lateral ventricle (arrow). (B) Axial CTA demonstrates a right medial frontal lobe AVM (arrow) with a superficial draining vein. (C) Axial T2-weighted MRI demonstrates the right medial frontal lobe AVM that extends into the SMA (arrow). Coronal (D) and sagittal (E) T1-weighted MRI showing the location of the lesion within the medial right frontal lobe (arrows). AVM: arteriovenous malformation; CTA: computed tomography angiography; CTH: computed tomography of the head; MRI: magnetic resonance imaging; SMA: supplementary motor area

We illustrate a case of a 21-year-old, previously healthy, right-handed female who presented after a sudden onset of headache and vomiting. Her Glasgow Coma Score was 15, and there were no focal deficits on neurological examination. Computed tomography of the head (CTH) demonstrated intraventricular hemorrhage that appeared to arise from the anterior horn of the right lateral ventricle (Figure 1A). Her intracerebral hemorrhage score was 1 for intraventricular hemorrhage [5]. Computed tomography angiography and magnetic resonance imaging (Figures 1B-1E) demonstrated a large medial right frontal lobe AVM. The lesion was in the region of the supplementary motor area (SMA) and cingulate gyrus.

**Figure 2:**
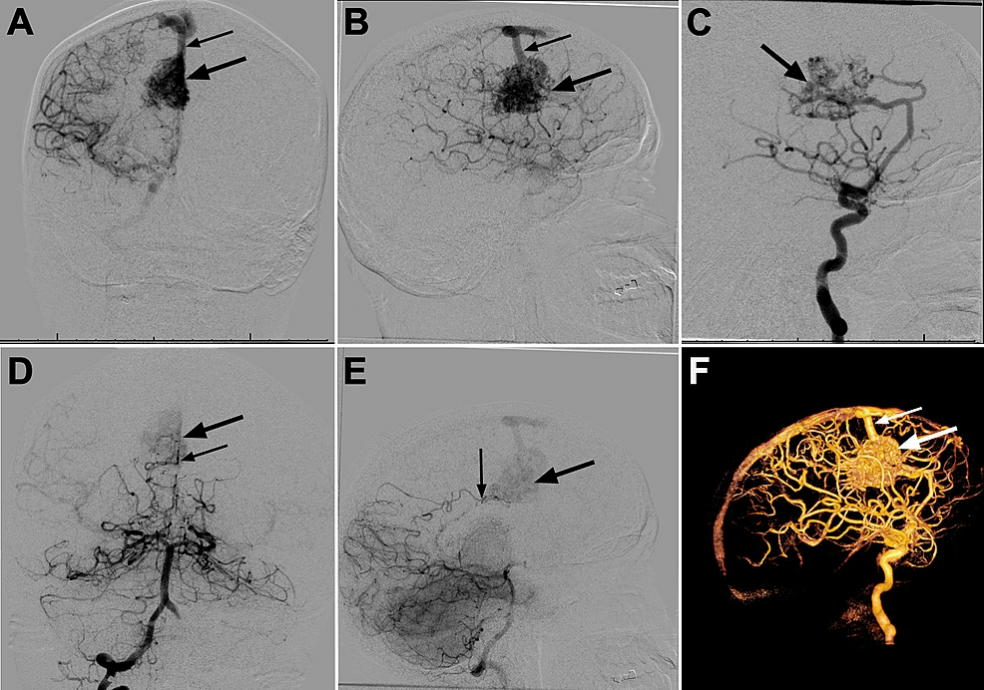
Preoperative DSA. (A) Anteroposterior and (B) lateral views of right internal carotid artery injection in the late arterial phase. These images demonstrate a medial right frontal nidus (large arrows) with superficial venous drainage emptying into the superior sagittal sinus (small arrows). There is no evidence of deep venous drainage. (C) Lateral view of right internal carotid artery injection in the early arterial phase demonstrating an intranidal aneurysm (arrow). Anteroposterior (D) and lateral (E) views of the right vertebral artery injection demonstrating arterial supply to the AVM (large arrows = nidus) from a distal right posterior cerebral artery branch (small arrows). (F) Lateral four-dimensional reconstruction of a right internal carotid artery injection. There is superficial venous drainage to the superior sagittal sinus without evidence of deep venous drainage (large arrow = nidus, small arrow = superficial draining vein). AVM: arteriovenous malformation; DSA: digital subtraction angiography

Further imaging with DSA demonstrated that the right frontal AVM nidus measured 36.8 × 25.4 mm and was supplied predominantly by the right pericallosal and callosomarginal arteries (Figures 2A, 2B). The right pericallosal artery and one of its larger branches were running en passage. Furthermore, the distal right callosomarginal artery appeared to empty directly into the AVM without evidence of en passage vessels. There was evidence of an intranidal aneurysm (Figure 2C), but this was not the source of the hemorrhage. The AVM also received a small arterial contribution from a distal right posterior cerebral artery branch (Figures 2D, 2E). There was a large nidal draining vein that extended superiorly along the right side of the cerebral falx draining into the superior sagittal sinus (Figure 2F). The lesion was classified as S-M grade II AVM (2 points for size).

**Figure 3:**
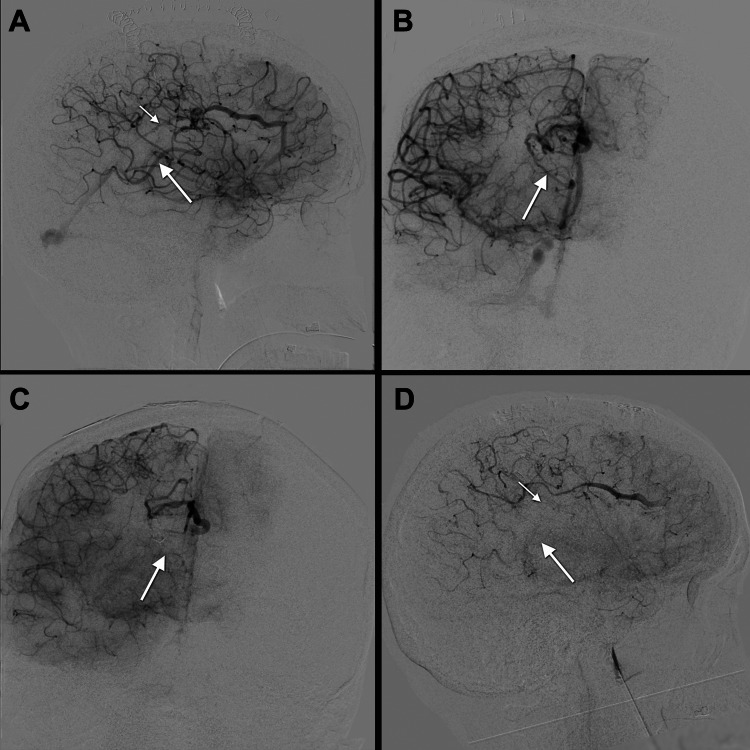
Postoperative DSA. (A, B) First postoperative DSA. (A) Anteroposterior and (B) lateral views of right internal carotid artery injection in the late arterial phase. Residual nidus can be seen along with deep venous drainage to the inferior sagittal sinus (small arrow in A) and additional deep venous drainage into the right basal vein of Rosenthal (large arrow in A and B). (C, D) Second postoperative DSA. (C) Anteroposterior and (D) lateral right internal carotid artery injection in the late arterial phase demonstrating complete resection of the AVM nidus and without evidence of arteriovenous shunting. The arrows demonstrate where the respective deep venous drainage used to reside. AVM: arteriovenous malformation; DSA: digital subtraction angiography

The patient remained stable preoperatively, and after discussing multiple therapeutic approaches, she opted for surgical resection. She underwent a right frontal craniotomy with an interhemispheric approach for surgical resection. After resection, which was thought to be a surgical cure, she remained under general anesthesia and underwent an immediate cerebral DSA. The DSA revealed residual nidus with two sources of deep venous drainage: one short draining vein entered the inferior sagittal sinus and another entered the right basal vein of Rosenthal (Figures 3A, 3B). Thus, the AVM was reclassified as S-M grade III. We believed we could safely resect the residual nidus; therefore, we returned to the operating room for additional resection. On DSA after additional resection, there was no evidence of residual arteriovenous shunting (Figures 3C, 3D).

**Figure 4:**
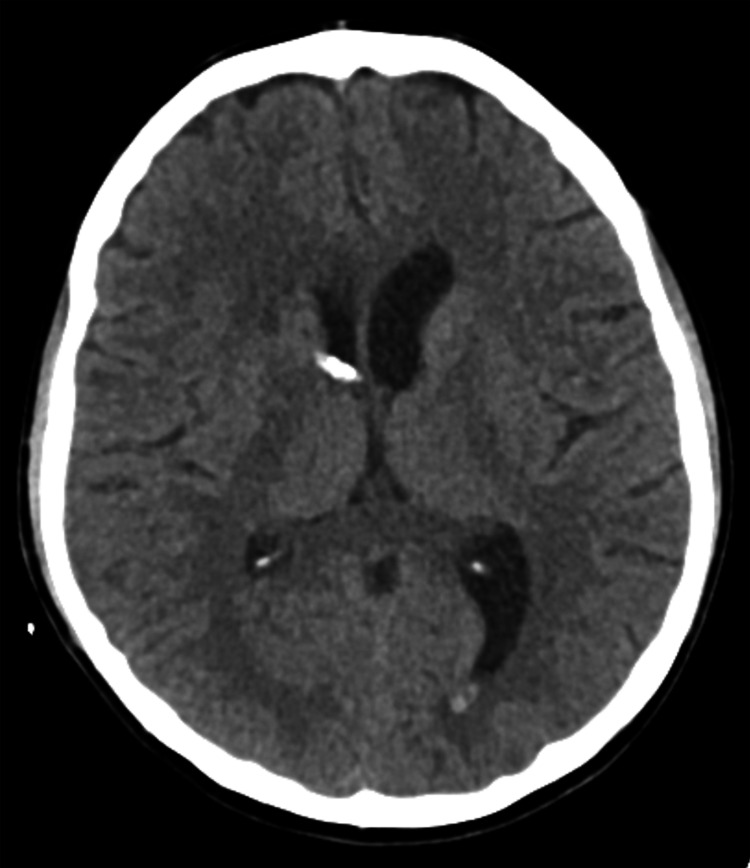
Imaging from postoperative day 11. The axial noncontrasted CTH demonstrates resolution of the intraventricular hemorrhage without evidence of infarction. CTH: computed tomography of the head

Postoperative CTH demonstrated expected postoperative changes that were stable on repeat imaging (Figure 4). On postoperative examination, she demonstrated antigravity resistance in her left upper and lower extremities with weakness most pronounced in her left upper extremity. Given the lesion’s involvement of the right SMA, this was thought to be SMA syndrome. The weakness continued to improve throughout her postoperative stay. After drainage of the remainder of the intraventricular hemorrhage, she was weaned from the external ventricular drain. She was discharged home on postoperative day 12 with outpatient physical, occupational, and speech therapies for rehabilitation. At her one-month follow-up appointment, she was neurologically intact with full resolution of her right-sided SMA syndrome. CTH at this time demonstrated expected postoperative changes and interval resolution of intracranial blood products, without evidence of hydrocephalus.

## Discussion

Cerebrovascular AVMs have an incidence rate of 1.42 cases per 100,000 person-years [6]. The presence of an AVM represents a significant risk of morbidity and mortality from intracranial hemorrhage, conferring a 2-4% annual risk of hemorrhage. If a hemorrhage has occurred, there is a 17-90% lifetime risk of rehemorrhage [1,7-10].

Surgical treatment of intracranial AVMs remains challenging, and judicious selection of microsurgical candidates remains a central challenge in predicting outcomes. The S-M grading scale considers the size, venous drainage, and eloquence of the involved brain region to give a numerical value between 1 and 5; it is the most commonly used grading system to assess surgical risk [9,11]. These grades are useful for directing treatment because a higher grade correlates with a greater risk of major deficit postresection and a lower grade correlates to a greater risk of hemorrhage [2,4,8,12,13].

Further studies have shown that outcomes and suggested treatment for grades I-II are similar, with resection recommended because of hemorrhage risk. For grade III AVMs, although there is a lack of randomized controlled trial data to guide management [13], there has been a trend toward multimodal approaches that combine the elements of stereotactic radiosurgery, endovascular embolization, and microsurgical resection [14,15].

In this case, we presented a patient who underwent microsurgical resection for what was initially believed to be a grade II AVM. This treatment modality was appropriate based on prior literature. This unique case demonstrates that the AVM was more accurately graded as an S-M grade III, although this information did not come to light until her first intraoperative DSA. We postulate that the deep draining veins were not seen on preoperative DSA because the superficial draining vein was large and drained most of the flow from the nidus, leaving the deeper veins with insufficient flow to be seen on DSA until after the resection with the subsequent sacrifice of the superficial draining vein and redirection of draining blood flow. Despite the presence of the deeper draining veins that required additional resection, this patient experienced an excellent outcome and complete treatment of her AVM.

## Conclusions

Postresection DSA is the gold standard for demonstrating a surgical cure for AVM. This case demonstrates the importance of considering immediate postoperative DSA under the same general anesthesia session and demonstrates deep venous drainage first seen on the immediate postoperative DSA. To our knowledge, this is the first documented case of AVM residual with new deep draining veins not visualized on preoperative DSA. Most notably, this upgraded the AVM from S-M grade II to III. Ultimately, the patient underwent surgical resection without long-standing neurological deficits and has experienced excellent recovery.
